# Prolonged Treatment Response to Pembrolizumab in a Patient with Pretreated Metastatic Colon Cancer and Lynch Syndrome

**DOI:** 10.1155/2019/3847672

**Published:** 2019-09-02

**Authors:** Matthew Keating, Lisa Giscombe, Toufic Tannous, Kevan Hartshorn

**Affiliations:** ^1^Department of Hematology/Oncology, Roger Williams Medical Center, Providence, RI, USA; ^2^Department of Hematology/Oncology, Boston University School of Medicine, Boston, MA, USA

## Abstract

Pembrolizumab and other immunotherapies now play a prominent role in the treatment of metastatic colon cancer. Clinicians have achieved significant response rates even in heavily pretreated patients, particularly those with mismatched repair deficiencies. The endpoint of pembrolizumab treatment for patients who enjoy a strong response remains unclear. Herein, we present the case of a 33-year-old man with pretreated metastatic colon cancer and a prolonged treatment response of over three years to single-agent pembrolizumab even after treatment discontinuation in July 2018. Prior to pembrolizumab, he was found to have lung and liver metastases despite multiple lines of chemotherapy. With pembrolizumab, there was a persistent downtrend in CEA level and uptrend in weight. After nearly three years of pembrolizumab treatment from October 2015 through July 2018, PET scan showed no FDG-avid disease, and further treatment was placed on hold. He remains under surveillance, with CT scan in February 2019 again showing no evidence of local or metastatic disease. In patients whose treatment duration and disease course are not defined by toxicities/progressive disease but rather by sustained treatment responses, we propose that immunotherapy treatment duration be guided by close monitoring of CEA levels, weight, and clinical exams in addition to traditional imaging.

## 1. Introduction

The role of immunotherapy in various malignancies continues to grow, and colon cancer is no exception. Pembrolizumab and nivolumab are two immune checkpoint inhibitors with key roles in various advanced malignancies including the lung, kidney, colon, and bladder as well as melanoma and lymphomas. These immunotherapies block the binding of programmed death-ligand 1 (PD-L1) to its receptor programmed death-1 (PD-1) on activated T cells, thereby stimulating the immune system and thus the antitumor response. Significant response rates even in heavily pretreated patients have led to expedited approval of both pembrolizumab and nivolumab monotherapies in colon cancer, in 2015 and 2017, respectively [1, 2]. Combination therapy with nivolumab and ipilimumab has also been approved since 2018 based on results from the phase II CheckMate 142 trial [3].

Mismatch repair deficiencies (dMMRs) are predictive for a response to immunotherapy in metastatic colorectal cancers. A mismatch repair deficiency allows for markedly increased numbers of deoxyribonucleic acid (DNA) mutations to pass unrepaired during cell division, thereby promoting tumor growth. There are four main mismatch repair genes including MLH1, MSH2, MSH6, and PMS2. Those with MLH1 and MSH2 mutations tend to develop colorectal cancer earlier relative to those patients with PMS2 mutations. MSH2 mutations are thought to place the patient at higher risk for extracolonic cancers relative to those with MLH1 mutations [4].

In hereditary nonpolyposis colorectal cancer (Lynch syndrome), an inherited germline mutation in one of the mismatch repair genes listed above coupled with a somatic mutation in the wild-type allele gives rise to dMMR. Lynch syndrome is responsible for 2-5% of colorectal cancers. In sporadic mismatch repair deficiency, both alleles are compromised by either somatic mutations or epigenetic silencing [5]. Not all patients with Lynch syndrome will go on to develop colorectal cancer over the course of their lifetime, and cancer risk profiles are thought to be specific to each mismatch repair gene. Note that those with Lynch syndrome are also at risk for other cancers such as prostate and endometrial cancers.

Patients with dMMR typically manifest as microsatellite instability-high (MSI-H) owing to the increased number of DNA mismatches that go unchecked. Immune checkpoint inhibitor use in metastatic colon cancer is limited to those with MSI-H or dMMR tumors; however, in the future, the treatment population may include those with high tumor mutational burden due to mutated DNA polymerases or other abnormalities [6, 7]. Patients identified as MSI-H/dMMR with metastatic colon cancer are candidates for immunotherapy assuming they do not have significant autoimmune disease history or other contraindications to preclude the use of immune checkpoint inhibitors. Herein, we present the case of a 33-year-old man with pretreated metastatic colon cancer who is enjoying an ongoing, prolonged treatment response of over three years to single-agent pembrolizumab even after treatment discontinuation in July 2018.

## 2. Case Presentation

A 33-year-old Caucasian man with asthma and active smoking history initially presented in 2012 with severe intermittent abdominal pain, constipation, and 50-pound weight loss over the past year. Family history was strongly positive for colon cancer in his mother (diagnosed at 44), three aunts (all died of colon cancer in their 20s), and his uncle. In December 2012, he underwent colonoscopy and a mass in the descending colon was identified which could not be traversed. A preoperative CEA level was not obtained. In January 2013, he underwent subtotal colectomy and ileoproctostomy for findings of nearly obstructing descending colon cancer with abdominal sidewall invasion and involvement of two loops of small bowel. Pathology showed stage IIIC pT4bN1bMX moderate to poorly differentiated colon adenocarcinoma with 5% signet cell differentiation, 7.5 cm in greatest dimension. Lymphovascular and perineural invasion were present, and 3 of 29 lymph nodes were positive for adenocarcinoma. Margins were negative. Microsatellite instability testing was positive with loss of MSH2 and MSH6 expression, and KRAS mutation was positive in codon 13. Ultimately, he was diagnosed with Lynch syndrome based on genetic testing done on peripheral blood DNA which showed a deleterious Y656X mutation in MSH2. It can be inferred that loss of MSH2 subsequently led to loss of expression of MSH6 secondarily [8]. Magnetic resonance and CT imaging were negative for distant metastases or pathologic lymphadenopathy at this time. He underwent chemoradiation treatment with capecitabine from February to March 2013. Treatment was prematurely terminated due to severe toxicities of diarrhea, dehydration, and fungal endophthalmitis. DPD mutation testing was negative. Another trial of capecitabine in July 2013 resulted in similar toxicities with the first cycle. His adjuvant chemotherapy regimen was switched to weekly 5-FU+leucovorin which he received from August to November 2013, with some dosing adjustments due to dehydration, elevated liver function tests and creatinine, and abdominal pain. PET CT scan in December 2013 showed pathologic lymphadenopathy of the left-sided paraortic, mesenteric, and external iliac lymph nodes. Subsequent CT scan in April 2014 showed further increase in size of these lymph nodes and a rise in CEA to 15 (versus 9 in November 2013). The next CT scan in October 2014 showed stable lymph node size, and he was lost to follow-up at this time due to loss of insurance coverage.

He reestablished care in May 2015 and was found to have metastatic colon cancer with a new liver lesion and pulmonary nodules as well as increased size of lymph nodes. These lesions were clinically consistent with unresectable metachronous liver and lung metastases. CEA level was 47. CEA continued to rise, and he lost more weight despite administration of weekly 5-FU from June to August 2015. Irinotecan at slightly reduced dose and bevacizumab were given from August to October 2015, but despite marked drop in CEA, he did not tolerate treatment well (diarrhea and elevated creatinine requiring hospital admission). UGT1A1 testing had previously shown positive heterozygous TA7 polymorphism. A further dose reduction was made to try to achieve a tolerable but still effective dose, but this was still complicated by diarrhea and, in addition, his CEA began to rise.

**Figure 1:**
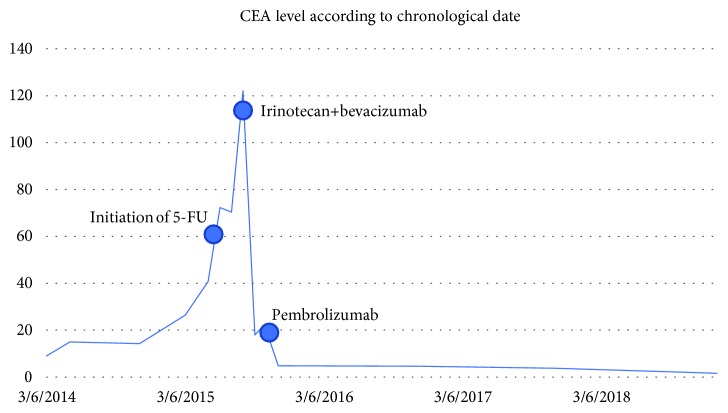
CEA level according to chronological date. The start dates of the patient's most recent chemotherapy and immunotherapy regimens are marked for reference.

**Figure 2:**
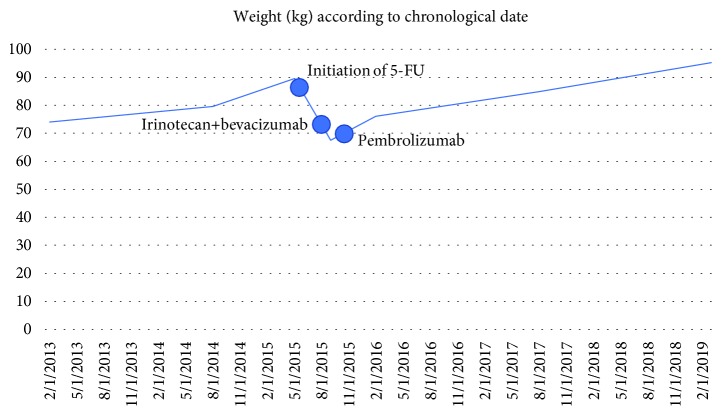
Weight according to chronological date. The start dates of the patient's most recent chemotherapy and immunotherapy regimens are marked for reference.

**Figure 3:**
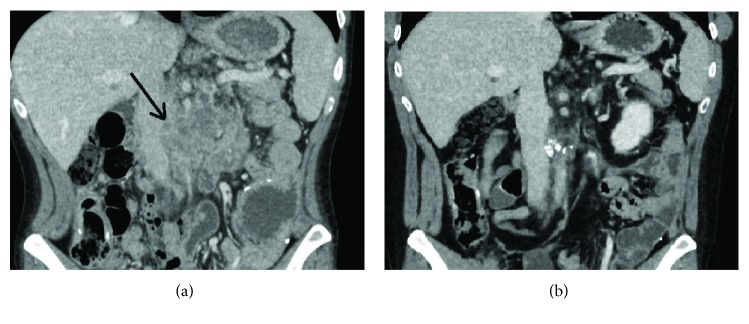
CT contrast imaging before (a) (October 8, 2015) and after (b) (February 1, 2019) administration of pembrolizumab. The black arrow in panel (a) indicates a large retroperitoneal lymph node conglomerate which compressed the inferior vena cava, encased the aorta, and invaded the left renal vein. The lymph node conglomerate has since been reduced to small dense calcifications as seen in panel (b).

In October 2015, he was started on pembrolizumab 2 mg/kg every 21 days, with a corresponding drop in CEA level and clinical improvement. CEA decreased to within normal limits, and his weight increased over the course of pembrolizumab treatment (Figures 1 and 2). He continued pembrolizumab treatment through July 2018, at which time, it was decided to implement a treatment break given stable disease with no FDG avidity on PET scan at that time (though small calcified residual lymph node findings were present). Aside from immunotherapy-induced hypothyroidism, he tolerated treatment well. He received a total of 48 cycles of pembrolizumab. CEA remains within normal limits and surveillance CT scan in February 2019 showed no evidence of metastatic disease, indicating a remarkable response when compared to the CT prior to the initiation of immunotherapy (Figure 3). He is being followed with yearly flex sigmoidoscopy, upper endoscopy every two years, and oncology clinic visits with CEA checks every six weeks.

## 3. Discussion

While durable responses to immunotherapy in dMMR metastatic colon cancer are not unusual, our patient's case is exceptional because he has enjoyed over three years and counting of progression-free survival, despite stopping pembrolizumab treatment over seven months ago. Leal et al. identified a median overall survival from pembrolizumab of 16.1 months in a retrospective cohort of 19 patients with dMMR metastatic colorectal cancer [9]. Most patients with dMMR metastatic colon cancer treated with immunotherapy will survive at least 12 months after the initiation of immunotherapy. One study did observe a large difference in overall response rate for patients with Lynch syndrome versus other forms of dMMR. Lynch syndrome patients had a 27-percent overall response rate (11 patients) versus 100-percent overall response rate (6 patients) in those with dMMR not linked to Lynch syndrome [5]. This discrepant result between the dMMR subgroups has not been duplicated in other studies to date to our knowledge, but if true, would make our Lynch syndrome patient's response all the more surprising. Furthermore, our patient remains in remission per normal CEA and surveillance imaging despite stopping pembrolizumab over seven months ago to allow for an extended treatment break. The endpoint of pembrolizumab treatment after a strong response remains unclear, and further research is needed to define treatment duration in this setting. Our patient showed excellent tolerance of immunotherapy despite suffering a high level of toxicity with standard chemotherapy and radiation. While there are many known immunotherapy-induced toxicities of PD-L1 inhibitors, these are generally well-managed when addressed early as with our patient's immunotherapy-induced hypothyroidism, and side-effects do not usually compromise the intended treatment course.

Immunotherapy's role in colorectal cancer may soon be refined beyond the dMMR and MSI-H populations. Anecdotal evidence and small retrospective analyses have been reported that link immunotherapy treatment response with high tumor mutational burden due to mutated DNA polymerases or other abnormalities, independent of mismatch repair status [6, 7]. DNA polymerase mutations are implicated in 1% of colorectal cancers and can even coexist with mutated mismatch repair genes [10]. A more in-depth molecular analysis of dMMR and MSI-H populations may hold the answer to why certain individuals enjoy prolonged immunotherapy responses as with our patient. Even if predictive significance remains elusive with future studies, the complete molecular profile has already been shown to hold prognostic significance. Our patient has coexisting KRAS mutation and MSI-H status. KRAS mutations are inversely associated with MSI, so our patient's combined abnormalities are not common. When viewed alone, KRAS mutations have had mixed prognostic findings, with some studies showing negative prognostic value and others showing unaffected disease-specific outcomes. However, combined molecular analyses have proven more instructive. One study evaluated disease-specific survival for KRAS and BRAF mutation status with MSI. The best prognostic group was KRAS and BRAF wild-type with MSI, then KRAS or BRAF mutant with MSI, followed by KRAS and BRAF wild-type with MSS, and lastly, KRAS or BRAF mutant with MSS [11]. It would appear that KRAS and MSI yield the most prognostic value when interpreted together, begging the question of what other underlying molecular abnormalities that are yet to be analyzed collectively may add to prognostic and predictive data.

We would like to call attention to the close correlation of CEA levels with immunotherapy response in our patient and in outside studies. Our patient's CEA levels rose in the setting of recurrent metastatic disease, continued to climb with 5-fluorouracil-based treatment, initially responded well to irinotecan+bevacizumab-based treatment, and then began increasing again to 23 after a dose reduction of irinotecan was made due to severe toxicity. The CEA promptly dropped to the normal range with pembrolizumab treatment (Figure 1). The chemotherapy treatment responses and CEA levels were in keeping with expectations that MSI patients tend to respond better to irinotecan than 5-fluorouracil. False elevations of CEA levels are known to occur, but the degree of elevation tends to reinforce suspicions of recurrent/metastatic disease particularly in patients who had elevated CEA levels prior to initial resection of locoregional disease. A retrospective analysis found half of elevated CEA levels to be false positives when presenting after R0 resections of locoregional colorectal cancer. However, false positives for CEA levels over 15 ng/ml were rare, and CEA levels exceeding 35 ng/ml all represented true positives [12]. CEA levels often correlate to clinical responses even after a single dose of immunotherapy and precede the corresponding radiographic responses by several months [13]. Radiographic changes may even appear to falsely indicate disease progression in the setting of immunotherapy, particularly when scans are performed prematurely. Certainly more studies are needed to determine the appropriate surveillance regimen for metastatic colorectal cancer patients on extended courses of immunotherapy. There are patients such as ours whose treatment duration and disease course are not defined by toxicities/progressive disease but rather by sustained treatment responses, and we would argue that in these cases, the treatment duration should be guided by close monitoring of CEA levels, weight, and clinical exams in addition to traditional imaging.
